# Dysexecutive syndrome in amnesic mild cognitive impairment: a multicenter study

**DOI:** 10.1186/s12883-016-0607-2

**Published:** 2016-06-04

**Authors:** E. Blanco Martín, I. Ugarriza Serrano, X. Elcoroaristizabal Martín, L. Galdos Alcelay, A. Molano Salazar, R. Bereincua Gandarias, S. Inglés Borda, J. M. Uterga Valiente, M. B. Indakoetxea Juanbeltz, J. Moraza Lopez, M. Barandiarán Amillano, M. Fernández-Martínez

**Affiliations:** Department of Neurology, Hospital Universitario Cruces, Barakaldo, Vizcaya Spain; BioCruces Health Research Institute, Barakaldo, Vizcaya Spain; Genetracer Biotech, Santander, Cantabria Spain; Hospital Universitario Álava, Vitoria-Gasteiz, Álava Spain; Department of Neurology, Hospital Universitario Basurto, Bilbao, Vizcaya Spain; Department of Neurology, Hospital Universitario Donostia, Donostia-San Sebastian, Guipúzcoa Spain; Biodonostia Health Research Institute, Donostia-San Sebastian, Guipúzcoa Spain

**Keywords:** Disexecutive syndrome, Executive functions, Amnesic mild cognitive impairment Alzheimer’s disease

## Abstract

**Background:**

Executive functions (EF) in Alzheimer’s disease (AD), classically related to the prefrontal cortex, have been forgotten in mild stages, given more importance to temporal lobe associated disorders, such as memory. The study of disexecutive syndrome (DS) has been relegated to advanced stages of the disease. Our goal is to demonstrate that EF are already present in amnesic mild cognitive impairment (aMCI). Furthermore, we are interested in knowing whether poor scores in EF tests are related to the progression to AD or another kind of dementia.

**Methods:**

We studied patients with aMCI (*n* = 81) and healthy controls (*n* = 142) from neurological departments of several centers of Basque Country with a cross-sectional design. Patients underwent a complete neuropsychological evaluation, neuroimaging testing APOE genotype and 3 year of prospective follow-up.

**Results:**

In the first visit, patients with aMCI showed more alterations in tests that evaluate EF such as Stroop, trail-making and categorical verbal fluency. More alterations were also found in NPI scale (*P* <0.05). Stroop and Trail-Making test were not associated with the future development of AD, but fluency (*p* = 0.01) and apathy (*p* = 0.031) did. No patient developed a different kind of dementia different from AD.

**Conclusions:**

DS is a broad concept not confined to frontal lobes, and can be found in early stages of aMCI. DS impacts negatively on patient autonomy and may have prognostic value.

## Background

Executive functions (EF), a term coined by Lezak [1] following the footsteps of Luria, are those mental capacities necessary for formulating goals, planning how to achieve them, and carrying out the plans effectively, encompassing tasks such as inhibition, interference control, working memory and cognitive flexibility. EF allow us to work with ideas, take time to think before acting, adapt to new and unexpected situations, avoid temptations and stay focused on something.

About the limits of EF located in prefrontal cortex, there is a certain lack of consensus and today its spectrum include not only cognitive aspects but also behavioral aspects. Impairment or loss of these functions compromises a person’s capacity to maintain an independent, constructively self-serving, and socially productive life [1, 2]. This is the case of Alzheimer disease (AD) [3]

Neurodegeneration in AD typically begins in the medial temporal lobe [4] leading to episodic memory deficits and to an inability to learn new information. As long as the disease progresses other brain areas will be impaired, producing language disorders, apraxia, visuospatial and executive disorders, and social and emotional dysfunctions. There are atypical forms of AD such as frontal variant, with a severe and disproportionate early alteration in the frontal functions and greater prominence of neurofibrillary tangles in the frontal cortex than in patients with classical Alzheimer’s disease [5].

However, in general terms we can face the model of the prototypical AD to frontotemporal dementia (FTD), in which degeneration begins in frontal and temporal lobes and the initial symptoms include deficits in executive functions and language, often with episodic memory relatively intact.

Many authors [6] believe that executive functions are relatively preserved in early stages of AD, being impaired later in the course of the disease, reflecting a moderate to severe cognitive impairment, related to the severity and duration of AD [7]. However, this is a controversial matter, and during the past 20 years, the literature has evolved and reported that AD patients were early impaired on executive functions [3, 8] especially those called “cold“ executive functions, because the corresponding cognitive processes tend not to have an emotional involvement and are relatively mechanical or logic. Unlike ”hot” executive functions in which emotions are more relevant, such as the experience of reward-punishment, the social behavior of each individual or the capacity of making decisions based on social and emotional aspects [9, 10].

Mild cognitive impairment (MCI), is a condition in which there are cognitive deficits involving specific domains in the absence of significant functional impact [11]. Current classifications recognize subtypes, depending on the presence or absence of impairment in episodic memory, denominating them amnestic mild cognitive impairment (aMCI) or non amnestic mild cognitive impairment. We can also subclassificate patients based on whether cognitive dysfunctions are single or multiple (single-domain, multi-domain) [12]. These classifications and sub-classifications may represent the prodromal situation of different types of dementia. According to this classification aMCI is often a previous stage of AD, and in one year 12 % of mild amnestic cognitive impairments will progress to AD [11].

Our study aims is to demonstrate that DS in AD is present in previous stages, such as aMCI. We also are interested in knowing whether the association of memory problems and executive impairment in aMCI leads to a different type of dementia such as FTD and to study the relationship between the scores of the EF and the progression from aMCI to AD.

## Methods

### Subjects

From a sample of 296 subjects recruited consecutively from the neurological departments of several centers of the Basque Country during one year period, we selected for this study only patients with aMCI (*n* = 81) and healthy controls (*n* = 142), the remaining 73 corresponded to patients with EA on the first visit.

And we followed up them yearly during a period of three years, using an extensive battery of tests and neuropsychological scales.

Neurologists with experience in cognitive impairment and neuropsychologists, who have previously worked together in other projects, evaluated all the patients and they used validated scales in Spanish in order to get inter-rater reliability:

- Mini-Mental State Examination (MMSE) [13, 14].
- Seven-minute neurocognitive screening battery [15, 16] including: a) Benton Orientation Test (temporal orientation), b) The Clock Test (visuospatial and visual-constructional capacity), c) Facilitating free recall (modification from the paradigm proposed by Buschke et. al. to evaluate episodic memory) and d) categorical verbal fluency (semantic memory and word retrieval strategy).
- Clinical Dementia Rating (CDR) for global assesment [17].
- Consortium to Establish a Registry for Alzheimer’s Disease (CERAD) [18] we assessed the list of words (memory), Trail-Making test parts A and B (attention and executive functions) and Boston test (language, nomination of images).
- Behavioral disturbances in patients were evaluated using the Neuropsychiatric Inventory (NPI) [19, 20] and disability by the Interview for Deterioration in Daily Living Activities in Dementia (IDDD) [21].
- Stroop A, B,C [22] (attention and executive functions)
- Ideomotor praxis were also systematically evaluated using Barcelona test [23].

EF were assessed by using Trail-making, Stroop and categorical verbal fluency test.

The results of these evaluations allow us to classify patients in different diagnostic groups.

The diagnosis of patients with aMCI was based on Petersen’s [24] and Winblad [25] criteria. Patients had memory complaints corroborated by an informant, representing a decline from a previous level of functioning given their age and educational level. The score in the CDR scale was required to be 0.5, and performance in relation to other cognitive functions and daily living activities was required to be normal.

Healthy control subjects were scored within the normal ranges for age and educational level in psychometric testing, with a CDR score of 0.

AD diagnosis was based on the DSM IV and NINCDS-ADRDA criteria for probable and possible AD

For patients with aMCI, evaluation also included routine blood tests: hematology, biochemistry, thyroid stimulating hormone, vitamin B12 levels, syphilis serology and neuroimaging test: CT scan or MRI. APOE genotyping was performed in all patients.

The exclusion criteria included: severe comorbidities making adequate follow-up unlikely, acute psychiatric diseases, previous cerebrovascular diseases (transient ischemic attacks, stroke or intracranial hemorrhage), other neurodegenerative diseases and the absence of a reliable informant.

### Records and informed consent

A specific database (Microsoft Access 2013) was designed and declared to the Spanish Data Protection Agency. The study was approved by the Ethics Committee of Cruces Hospital (Barakaldo, Spain). All patients signed informed consent to undergo the examination. The study was conducted in accordance with the Declaration of Helsinki concerning medical research in human subjects.

### Design of the study

In this cross-sectional study, we analyzed in the first visit the scores from each group of patients: Control and aMCI group (diagnostic category) and progression and stable group (progression category, defined by the future clinic progression or not of controls to aMCI and of aMCI to AD during the follow up period).

### Statistical analysis

Data were analyzed using SPSS 22v. for Windows.

In the first visit demographic variables between groups (aMCI and controls) and the test scores were studied, in order to determine if there were early differences. The independent variable was the diagnostic category (aMCI and controls) and the dependent variable the scores of the test. As the data were not normally distributed, to establish the differences, we used non-parametric test: U of Mann–Whitney and W of Wilcolson.

Then, we focused in the first visit of aMCI patients and we studied if EF, besides other factors (sex, age at first visit, years of schooling, APOE genotype and scores of other test than those evaluating EF) could be related to the progression of the disease. Now the independent variable was the presence or absence of clinical progression. Quantitative dependent variables (Test scores, age at first visit and years of schooling) were analyzed using U of Mann–Whitney and W of Wilcolson and qualitative variables (sex and presence or absence of APOE ε4 genotype) using chi-square. For those significant or close to the signification values we applied a model of binary logistic regression to perform a multivariate analysis.

A correlation analysis was also done (Pearson Correlation Coefficient) between scores that measure EF and IDDD, as well as between EF and apathy and memory in MCI.

## Results

There were no differences in age and sex between patients with cognitive impairment and controls. There were differences in years of schooling: higher values were found in controls than in aMCI (Table 1). Controls and aMCI also differ in MMSE scores, clock test, IDDD and NPI scale (*p* <0.01 in agitation, depression, anxiety, apathy, irritability and in changes in appetite). There was no difference in unilateral and bilateral praxis.

**Table 1:** Summary of baseline characteristics of study participants

	Controls (*n* = 142)	aMCI (*n* = 81)	*P* value
Women	80 (56.3 %)	42 (51.9 %)	0.58
Men	62 (43.7 %)	39 (48.1 %)	
schooling	9.48 (4.87)	8.20 (4.16)	**0.02**
Age First visit	70.97 (8.43)	71.51 (7.14)	0.69
3 years progression	6 (4.2 %) ^a^	21 (25.9 %)^b^	**<0.01**
APO ε4 alele	34 (23.9 %)	29 (35.8 %)	0.09
MMSE	28.1 (1.82)	26.51 (2.51)	**<0.01**
Denomination	13.41 (1.97)	12.21 (2.36)	**<0.01**
Clock test	6.28 (1.34)	5.65 (1.59)	**<0.01**
Unilateral Praxis	15.60 (0.89)	15.45 (1.19)	0.65
Bilateal praxis	7.05 (1.35)	6.80 (1.50)	0.123
IDDD	33 (0.25)	37.30 (4.67)	**<0.01**
NPI total	0.4 (1.04)	5.53 (7.12)	**<0.01**

^a^Progression to aMCI, ^b^Progression to EA. Mean (standar deviation)

Note: Values that are statistically significant are indicated in bold

Regarding the scores for the EF test between controls and aMCI (Table 2), there were statistically significant differences in Stroop A, Stroop B, Stroop C, Trail-Making A time, in addition to all Trail-Making B items.

**Table 2:** In aMCI. Comparison between EF test scores

Test	Control	aMCI	*P* value
Stroop A	86.89 (18.53)	75.46 (18.61)	**<0.01**
Stroop B	57.12 (12.42)	48.99 (14.48)	**<0.01**
Stroop C	28.91(9.75)	21.89 (11.15)	**<0.01**
Trail Making A Items	-	24.85 (1.31)	0.19
Trail Making A time	62.74 (32.45)	83.93 (34.59)	**<0.01**
Trail Making A mistakes	0.17 (0.86)	0.23 (0.61)	0.13
Trail Making B items	23.11 (4.57)	19.45 (7.32)	**<0.01**
Trail Making B time	128.05 (48.10)	166.49 (43.75)	**<0.01**
Trail Making B mistakes	0.75 (1.24)	1.69 (1.94)	**<0.01**
Verbal fluency	18.71(5.98)	14.68 (5.13)	**<0.01**

Mean (standard deviation)

Note: Values that are statistically significant are indicated in bold

During the three year period of follow up, completed by 57 % of controls and 63 % of aMCI, 6 patients (4 %) progressed from control to aMCI and 21 patients (26 %) progressed to AD, nobody progressed to dementia from control group.

We also analyzed if there were differences in the EF test scores between those patients with aMCI who experience clinical progression to dementia compared to those who remained stable (Table 3). There were no statistically significant differences in Trail-Making and STROOP test (although STROOP C *p* = 0.082) but we found differences in verbal fluency (*p* <0.01).

**Table 3:** Progression vs stable, differences between EF test scores

	Progression	Stable	*P* value
Stroop A	70.72 (18,03)	77.08 (18.70)	0.22
Stroop B	48.11 (16.51)	49.28 (13.89)	0.84
Stroop C	19.17 (12.73)	22.81 (10.53)	0.08
Trail Making A Items	-(−)	24.79(1.51)	0.55
Trail Making A time	82.17 (32.90)	84.53 (35.44)	0.81
Trail Making A mistakes	0.17 (0.71)	0.25 (0.59)	0.67
Trail Making B items	21.78 (5.77)	18.66 (7.67)	0.10
Trail Making B time	169.39 (29.27)	165.51(47.88)	0.74
Trail Making B mistakes	1.56 (1.10)	1.74 (2.16)	0.60
Verbal fluency	12.28 (4.66)	15.49 (5.06)	**0.01**

Note: Values that are statistically significant are indicated in bold

On the other hand, we assessed if there were other conditions that may influence the clinical progression, such as sex, age at first visit, years of schooling, apoE4 genotype or scores on memory tests, clock test or NPI scale (Table 4). Focusing on these factors that may influence the progression we found significant differences in APOE 4 genotype (*p* = 0.02), so those with at least one AP0E4 positive allele have a higher risk of progressing to dementia, OR of 3,294 (95 % CI 1,174- 9,243). No differences were found in gender. There were also significant differences in Facilitating free recall, list 2 and 3 and delayed recall of the list 1 of CERAD. Regarding the NPI Scale the total sum of all items has offered no meaningful information, but the analysis item by item did, and apathy reached a significance level of 0.031.

**Table 4:** Other factors that may influence the progression

	Progression	Stable	*P* value
Schooling	10.83 (5.28)	7.77 (3.50)	0.26
First visit age	72.17 (5,37)	70.87 (7,79)	0.66
Women	21.43 %	78.57 %	0.338
Men	30.77 %	69.23 %	
APOE 4 (+)	41.38 %	58.62 %	**0.02**
APOE4 (−)	17.65 %	82.35 %	
MMSE	26.39 (1.82)	26.77 (2,63)	0.11
NPI total	6.64 (7.46)	5.14 (7.02)	0.24
Apathy (NPI)	2.58 (2.87)	1.21 (2.37)	**0.031**
Denomination	11.57 (3.06)	12.44 (2.04)	0.376
Clock test	5.42 (1.80)	5.70 (1.50)	0.32
Facilitating-free recall	11.81 (3.37)	1379 (2.91)	**0.01**
CERAD list 1	2.29 (1.06)	2.98 (1.42)	0.05
CERAD list 2	4.00 (1.30)	4.66 (1.46)	**0.04**
CERAD list 3	4.48 (1.33)	5.76 (1.56)	**<0.01**
Recall list 1 CERAD	2.10 (1.76)	3.16 (1.99)	**0.04**
Recognition CERAD	16.05 (2.89)	16.69 (2.75)	0.39

Mean (estándar deviation)

Note: Values that are statistically significant are indicated in bold

Multivariate analysis of significative variables with binary logistic regression determine that only recall of the list 1 of CERAD remains significative (*p* = 0.01).

There was a discrete correlation (*p* = 0.01, *r* = 0.31) between the time spent in the performance of Trail Making B and IDDD scale. Correlations between memory tests, apathy and EF test in MCI showed also significant but subtle associations (Table 5).

**Table 5:** Correlations between memory test, EF test and apathy

		Fluency	Stroop A	Stroop B	Stroop C	TM A items	TM A time	TM A mistakes	TM B items	TM B time	TM B mistakes	Apathy
Buschke Mod.	Denomination	NS	NS	*r* = 0.23 *p* = 0.04	*r* = 0.24 *p* = 0.03	NS	*r* = −0.32 *p* = <0.01	*r* = −0.34 *p* = <0.01	NS	NS	NS	NS
	Immediate recall	*r* = 0.33 *p* < 0.01	NS	NS	*r* = 0.31 *p* < 0.01	*r* = 0.23 *p* = 0.04	NS	NS	*r* = 0.23 *p* = 0,05	NS	*r* = −0.42 *p* < 0.01	NS
	free recall	*r* = 0.43 *p* < 0.01	NS	*r* = 0.25 *p* = 0.03	*r* = 0,24 *p* < 0.01	NS	*r* = −0.43 *p* < 0.01	NS	*r* = 0.41 *p* < 0.01	NS	*r* = −0.33 *p* = 0.01	NS
	Facilitating recall	NS	NS	NS	*r* = 0.23 *p* = 0.46	NS	NS	NS	NS	NS	NS	NS
	Facilitating -free recall	*r* = 0.44 *p* < 0.01	*r* = 0.24 *p* = 0.03	NS	*r* = 0.47 *p* < 0,01	NS	*r* = −0.34 *p* < O.01	NS	*r* = 0.33 *p* < 0.01	NS	*r* = −0.26 *p* = 0.03	NS
CERAD List of word	List 1	*r* = 0.23 *p* = 0.04	NS	NS	NS	NS	*r* = −0.40 *p* < 0.01	NS	NS	*r* = − 0.26 *p* = 0.03	NS	NS
	List 2	*r* = 0.34 *p* < 0.01	r 0.25 *p* = 0.02	*r* = 0.36 *p* < 0.01	*r* = 0.30 *p* = 0.01	NS	r −0.27 *p* < 0.01	r-0.24 *p* = 0,03	NS	r- 0.29 *p* = 0.01	r-0.25 *p* = 0.01	NS
	List 3	*r* = 0,39 *p* < 0.01	*r* = 0.25 *p* = 0.02	*r* = 0.36 *p* < 0.01	*r* = 0.30 *p* = 0.01	NS	*r* = −0.45 *p* < 0.01	NS	NS	*r* = −0.30 *p* = 0.01	*r* = −0.30 *p* = 0,.01	*r* = −0.28 *p* = 0.02
	Recall list 1	*r* = 0.38 *p* < 0.01	*r* = 0.26 *p* = 0.02	*r* = 0.23 *p* = 0.04	*r* = 0.33 *p* < 0.01	NS	*r* = −0.52 *p* < 0.01	NS	*r* = 0.32 *p* = 0.01	*r* = −0.31 *p* = 0.01	*r* = −0.33 *p* = 0.01	NS
	Recognition	*r* = 0.23 *p* = 0.04		*r* = 0.32 *p* = 0.01	*r* = 0.35 *p* < 0.01	NS	*r* = −0.46 *p* < 0.01	*r* = −0.29 *p* = 0.01	*r* = 0.30 *p* = 0.01	NS	NS	NS

*NS* no significant

During the follow-up none of the 27 people (controls or aMCI) who progressed showed symptoms suggestive of other types of dementia different from Alzheimer’s disease.

## Discussion

The results of our study, in agreement with previous ones, [26–31] show that executive changes, measured in our case by Stroop test, trail-Making (Part B more relevant than A), are a very early finding in the course of the disease; This changes are present in aMCI.

Some authors [27, 28, 32, 33] point out the beginning of the executive impairment in mild stages of AD, specifically after the failures in episodic memory and before changes in language and cognition. Considering them the second most common neuropsychological impairment in mild AD followed by memory failures [3].

It should be mentioned to illustrate this precocity of appearance, one study [34] in which patients with subjective memory complaints (without mild cognitive impairment, dementia or other neurological or psychiatric disease) with confirmation of preclinical AD through the analisis of CSF, after two years of follow up began to show a decline in memory and executive functions.

Some of the most widely used tests of executive abilities, are the Wisconsin Card Sorting Task (WCST), the Stroop Test, and the Trail Making Test [28, 35]. But in order to increase the sensitivity to detect a dysexecutive syndrome and bring the assessment to the conditions of real life, it was developed the *Behavioral Assessment of Dysexecutive Syndrome* (BADS) [36], tool used to confirm the occurrence of disturbances in executive functions in aMCI and early AD, asserting that in most of the patients were altered other aspects besides memory. However sometimes deficits no related to memory are difficult to detect in a brief cognitive assessment.

Another recently developed tool for the assessment of cognitive and behavioral executive functions is the GREFEX battery. Used in one study whose authors said that up to 80 % of mild to moderate AD patients have alterations in EF. This battery includes verbal fluency which can be measure EF, but there is a lack of consensus on whether it should be considered within this group or not, in fact it is difficult to frame this dysfunction as a linguistic, semantic memory, dysexecutive alteration, or a deficit in processing speed [8, 37].

Apathy is included as a hallmark of the dysexecutive behavior, around which have emerged previous works relating it to the progression from MCI to AD and a thinning of the temporal cortex in aMCI [38–40].

Our study has highlighted the importance of measuring verbal fluency, through categorical evocation of animals. Not only because it is early altered in aMCI, but also because unlike the Trail Making or Stroop, it has been observed that there is an association between the scores of fluency and the progression to dementia. Indeed, previous studies showed a high sensitivity of verbal fluency test for detecting dementia. Recent prospective neuropsychological studies indicate that cognitive assessment can be an excellent indicator of future progression of MCI to AD particularly when episodic memory is combined with alterations in executive control and language tasks [41, 42].

With regard to disease progression, the available literature suggests that early onset of executive alterations favors the progression from MCI to AD [10, 32, 41–44]. In our study we have not been able to prove this assumption through the scores of Stroop and Trail-Making, This lack of significance may be explained because only 21 aMCI progressed to dementia during the three years follow-up at and it could be difficult to reach an statistical significance.

In our study, no distinction has been made between single-domain aMCI or multi-domain, but other authors [32] classified MCI patients into four subgroups (amnestic versus non amnestic, and single-domain versus multiple-domain. Planning/problem-solving and working memory, but not judgment, were impaired in the four subtypes, even among those with a pure amnestic MCI. Multiple-domain MCI patients had more severe impairments in planning/problem-solving and working memory than single-domain patients, leading to the supposition that they are at highest risk of imminent dementia comparing with not pure amnestic MCIs.

Regarding the impact of EF alterations in patient autonomy, several publications have highlighted their importance in real life situations such as handling money or taking regular medication, with a normal shallow cognitive assessment [2, 28]. Our data provides a significant correlation between IDDD scale and the time of the Trail-making type B.

Another discussed aspect is the anatomical basis of executive functions. Frontal lobe and EF have been traditionally considered as synonymous. In fact neuroimaging studies have demonstrated the involvement of the frontal lobe in “cold” executive tasks, with a homogeneous involvement of the prefrontal cortex [45]. However, recent data have suggested that “cold” executive functions are distributed over a wide cerebral network which includes posterior associative cortices [46], subcortical structures and thalamic ways. Some authors [47] have even linked cerebellar lobes with executive functions including working memory, multitasking and inhibition.

Currently, a great deal of attention is being paid to the study of EF and their relationship with the white matter, and the cortico-cortical brain networks, altered during the neurodegenerative process of Alzheimer’s disease [48]. Using advanced imaging techniques such as diffusion tensor, it has been confirmed the involvement of frontal and posterior brain areas (parietal lobes) in patients with MCI and impaired EF [49]. In addition to cingular alterations [50].

Another object of analysis in this study is whether there is an association between episodic memory and EF. In our study Buschke modified test or the CERAD’s list of words are good predictors of progression of the disease.

In AD executive deficits could be explained for the memory impairment. Some authors [6] suggest that failure in executive function reflects a selective vulnerability within limbic–cortical networks secondary to a temporal lobe dysfunction. Other authors [8] also detected significant correlations in mild AD and not in very mild cases between EF and memory. An association between verbal fluency with long term memory, but not with short term memory has also found. In addition, many of the proposed neuropsychological tests to evaluate executive functions also evaluate attention [51]. Attention is a complex higher brain function indispensable to memorize, and this fact may be possible explanation for these correlations.

The strengths of our study are its multicenter nature, the correct inclusion of patients into diagnostic categories, the representativeness of the sample and the broad battery of neuropsychological tests performed.

Our study design allow us an adequate characterization of patients and the prospective follow-up provides more information about the factors associated with the progression of cognitive impairment.

Aspects that can have a direct application in routine clinical practice, optimizing resources and to providing data to support prognostic informations.

This study represents a comprehensive approach to the dysexecutive syndrom in aMCI with the last informations published the last years.

Some limitations in our study must be addressed. Our population comes from a hospital setting. A community-based study could provide more information. Besides, the small number of patients who progress from MCI to AD (consistent with previous papers) makes difficult to draw conclusions. We have also used the most common tests for EF, however currently there are extensive batteries that includes not only cognitive but also behavioral aspects.

## Conclusions

Dysexecutive syndrome is a part of a set of alterations that can accompany episodic memory disorder in aMCI, its relevance lies in the negative impact on patient autonomy and its role as a predictor of the future development of dementia.

In our study we have only been able to prove this premise for the loss of categorical verbal fluency and apathy. We consider efficient to include systematically an assessment of both in the routine evaluation of a patient with aMCI.

Regarding the relationship between episodic memory and EF our data point out to a relationship between them, but further studies would be required to confirm this statement.

## Abbreviations

AD, Alzheimer’s disease; aMCI, Amnesic mild cognitive impairment; DS, Disexecutive síndrome; EF, Executive functions; FTD, Frontotemporal dementia.
